# Dipeptidyl Peptidase 10 (DPP10_**789**_): A Voltage Gated Potassium Channel Associated Protein Is Abnormally Expressed in Alzheimer's and Other Neurodegenerative Diseases

**DOI:** 10.1155/2014/209398

**Published:** 2014-06-16

**Authors:** Tong Chen, Wei-Ping Gai, Catherine A. Abbott

**Affiliations:** ^1^School of Biological Sciences, Flinders University, GPO Box 2100, Adelaide, SA 5001, Australia; ^2^Department of Human Physiology, School of Medicine, Flinders University, GPO Box 2100, Adelaide, SA 5001, Australia

## Abstract

The neuropathological features associated with Alzheimer's disease (AD) include the presence of extracellular amyloid-*β* peptide-containing plaques and intracellular tau positive neurofibrillary tangles and the loss of synapses and neurons in defined regions of the brain. Dipeptidyl peptidase 10 (DPP10) is a protein that facilitates Kv4 channel surface expression and neuronal excitability. This study aims to explore DPP10_789_ protein distribution in human brains and its contribution to the neurofibrillary pathology of AD and other tauopathies. Immunohistochemical analysis revealed predominant neuronal staining of DPP10_789_ in control brains, and the CA1 region of the hippocampus contained strong reactivity in the distal dendrites of the pyramidal cells. In AD brains, robust DPP10_789_ reactivity was detected in neurofibrillary tangles and plaque-associated dystrophic neurites, most of which colocalized with the doubly phosphorylated Ser-202/Thr-205 tau epitope. DPP10_789_ positive neurofibrillary tangles and plaque-associated dystrophic neurites also appeared in other neurodegenerative diseases such as frontotemporal lobar degeneration, diffuse Lewy body disease, and progressive supranuclear palsy. Occasional DPP10_789_ positive neurofibrillary tangles and neurites were seen in some aged control brains. Western blot analysis showed both full length and truncated DPP10_789_ fragments with the later increasing significantly in AD brains compared to control brains. Our results suggest that DPP10_789_ is involved in the pathology of AD and other neurodegenerative diseases.

## 1. Introduction

Alzheimer's disease (AD) is a progressive neurodegenerative disorder characterized pathologically by the presence of extracellular amyloid plaques containing the amyloid-*β* peptide (A*β*) and intracellular neurofibrillary tangles (NFTs) containing hyperphosphorylated microtubule-associated protein tau and loss of synapses and neurons in select brain regions [1–3]. The aetiology of AD is complex, with a majority of cases being sporadic and approximately 10% being inherited. Mutations have been identified in genes encoding amyloid precursor protein (APP) [4, 5], presenilin 1 (PS1), and PS2 in a small subset of familial AD cases. In sporadic AD cases, polymorphisms of apolipoprotein E4 and other genes have been associated with an increased risk of developing the disease [6].

Oxidative stress, impaired energy metabolism, and disruption of neuronal Ca^2+^ regulation have all been implicated in the cellular pathophysiology. Although experimental approaches indicate that A*β* deposition plays an important role in the neurodegenerative process in AD [7, 8], the mechanism underlying the relentless progression of neurofibrillary pathology and neuronal death remains to be fully elucidated. Over the last decade there have been a number of studies demonstrating that potassium channel dysfunction may be involved in the pathogenesis of AD [9–13].

Neuronal excitability is finely controlled by various membranous channels and associated proteins. In the central nervous system, the rapidly inactivating voltage-gated potassium channels (Kv channels) are the major determinants of dendritic excitability [14]. In particular, Kv4 channels, a subfamily of Kv channels, account for a large portion of the somatodendritic inactivating current in neurons in regulating firing frequency and signal processing in dendrites. The Kv4 channels are composed of the pore-forming subunits, various auxiliary subunits, and other interacting proteins such as Kv channel interacting proteins and dipeptidyl peptidase-like proteins DPP6 and DPP10 [15–17].

Indeed, potassium channel dysfunction has been demonstrated in fibroblasts and platelets of AD patients [9, 10]. Postmortem studies have also showed alterations of potassium channel expression in AD brains [18]. Several publications have demonstrated that A*β* is involved in Kv channel function and may even play a physiological role in controlling neuronal excitability [19, 20]. In addition, Kv channel interacting protein 3, also known as calsenilin, is a presenilin binding protein [21] that preferentially interacts with the familial AD associated *C*-terminal fragment of PS2 [22]. Moreover, the application of A*β* peptide to cultured cells increases both Kv channel interacting protein 3 mRNA and protein expression as well as cell death. The A*β* toxicity can be prevented by blocking expression of Kv channel interacting protein 3 [23]. Furthermore, in a study using a transgenic model of Alzheimer's disease, some potassium channels in hippocampal neurons were found to be absent [24]. Liu et al. reported that a potassium channel activator diazoxide ameliorated A*β* and tau pathologies and improves memory in the 3xTg AD mouse model [25]. Thus there is a body of research that suggests that potassium channels or their associated proteins might be involved in steps leading to the neurodegeneration observed in AD.

DPP10 belongs to the dipeptidyl peptidase 4 (DPP4) gene family sharing 59% amino acid sequence similarity with DPP4, an atypical serine protease [26, 27]. However DPP10 is missing the nucleophilic Ser residue in the catalytic motif and lacks enzyme activity. So far, four splice variants have been reported for DPP10. As a result of alternate splicing of the first exon, each variant has a short divergent cytoplasmic *N*-terminus. The remaining protein of each variant is identical and consists of a highly conserved juxtamembrane, transmembrane, and large extracellular *C*-terminal domain. Kv4 channels containing different DPP10 variants display distinct inactivation kinetics and voltage dependence at depolarized potentials, indicating that the cytoplasmic portion of the DPP10 protein influences the inactivation properties [28]. We have previously cloned the short form of DPP10 (DPP10_789_) from human brain [27]. To further characterise its function, we generated an antibody specific to DPP10_789_ to examine the expression of DPP10_789_ in human brains. Here we report that DPP10_789_ is predominantly localized to neuronal soma and dendrites in the neocortex and subcortical grey matter in control brains and abnormally accumulated in Alzheimer's disease and other diseases characterised by tau positive pathologies.

## 2. Materials and Methods

### 2.1. Primary Antibodies

The peptide H-MRKVESRGEGGRE-OH containing the *N*-terminus of DPP10_789_ was ordered from MIMOTOPES (VIC, Australia). This *N*-terminal sequence shares no sequence identity with the two other forms of DPP10 or the *N*-termini of the four DPP6 isoforms and therefore will only detect the DPP10_789_ isoform of DPP10 [29]. Rabbits were immunised by the peptide mixed with Freund's complete adjuvant. DPP10_789_ antiserum was purified using an affinity column prepared from the respective immunising peptide (ABGENT, Inc., San Diego, CA, USA) conjugated to 6% cross-linked beaded agarose. A sequence homology search was performed using BlastP into the GenBank database and the peptide sequence only had significant homology with DPP10_789_. All other primary antibodies, their source, and the dilution used in the present studies are listed in Table 1.

### 2.2. Human Cases

Samples from the hippocampus, the frontal and temporal cerebral cortex, the entorhinal and cingulated cortex, and the cerebellum were obtained from individuals listed in Table 2. Brain tissue was collected by the National Health Medical Research Council (NHMRC) Brain Bank of South Australia following NHMRC ethical guidelines. All diseased cases had a clinical diagnosis while control cases showed no clinical signs of dementia.

### 2.3. Cell Culture and Sample Preparation

The human kidney epithelial cell line 293T was cultured as described previously [30]. Transfection with GFP-DPP10_789_ construct was carried out by methods previously described using Fugene 6 (Roche, USA) [27, 30]. After 48 hrs, cell extracts were resuspended in sonication buffer (20 mM Tris pH 7.5, 5 mM EGTA, 2 mM EDTA and 0.25 M sucrose) and sonicated for 3 × 10 sec. Following sonication cellular debris was removed by a brief 10,000 g centrifugation and then the supernatant underwent a 30 min 100,000 g centrifugation at 4°C to pellet membrane proteins. The membrane pellet was solubilized in sonication buffer containing 0.5% Triton X-100, mixed with 4x sample buffer (6% SDS, 40% sucrose, 150 mM Tris pH 6.8, and 0.03% Bromophenol Blue), and incubated at 37°C for 10 mins and run on 10% SDS-PAGE. The recombinant GFP-tagged DPP10_789_ protein was detected using either an anti-GFP antibody (Cell Signaling Technology, USA) or the affinity purified anti-DPP10_789_ antibody described above.

### 2.4. Tissue Preparation

Brain tissues were fixed routinely by immersion in a mixture of 4% paraformaldehyde and picric acid. Series of each frozen section (450 *μ*m thick) were cut on a chilled microtome. Paraffin sections were cut (5–20 *μ*m), mounted on gelatine coated glass slides, and dried in an oven at 37°C overnight.

### 2.5. Immunohistochemistry

A green fluorescent protein GFP-DPP10_789_ construct with GFP fused to the *N*-terminus of DPP10_789_ was transiently transfected into 293T cells cultured on a chamber slide as described previously [27]. After 48 h, cells were washed with PBS and then fixed with 2% paraformaldehyde made in PBS. The affinity purified anti-DPP10_789_ Ab was used to detect recombinant DPP10_789_ protein. Mouse anti-rabbit Alexa 596 (Molecular Probes, Eugene, OR) was used as the secondary antibody for immunofluorescence studies.

Free-floating sections of human brain (50 *μ*m) were incubated with primary antibody for 48 h at 4°C. Incubation with the secondary biotinylated anti-rabbit or anti-mouse IgG was performed for 1 hr at room temperature. Immunoreactions were visualised with the avidin-biotin-peroxidase complex (VECTASTAIN ABC system, Vector Labs, UK) and 3-3-diaminobenzidine-4 HCl/H_2_O_2_ (DAB, D5637 Sigma, USA). Paraffin-embedded sections were treated similarly after dewaxing, except that they were heat-treated (105°C for 10 min 1 mM EDTA, pH 8.0 at 25°C) before immunostaining.

Double-labelled immunohistochemistry was performed on sections from AD cases. Selected free-floating sections were incubated with both anti-DPP10_789_ and AT8 antibody (anti-phosphorylated tau protein), Tau2 antibody (detects both phosphorylated and nonphosphorylated tau), or A4 antibody (anti-amyloid-*β*). Anti-DPP10_789_ antibody was then visualized by anti-rabbit IgG conjugated with Cy2 (Jackson ImmunoResearch Laboratories Inc., USA) while anti-tau antibody or anti-amyloid antibody was detected by anti-mouse IgG conjugated with Cy3 (Jackson ImmunoResearch Laboratories).

Whenever the A4 antibody was used, antigen retrieval was performed before the blocking step by incubating the sections in preheated sodium citrate buffer (10 mM, pH 8.5) at 80°C for 30 min.

### 2.6. Quantification of Lesions

Adjacent free-floating sections of hippocampus or cortex were immunostained with DPP10_789_, AT8, or A4 antibody separately using the DAB reaction as described above. The number of lesions was quantified at 200 times of magnification under an Olympus BH-2 light microscope (Tokyo, Japan) coupled to a MicroPublisher 3.3 RTV digital camera (QIMAGING, Canada). In the hippocampus, three fields (0.01 mm^2^/field) from each region of Ammon's horn (CA1-4) were counted. In neocortical sections, three fields per case were counted in each of layers III to V. After each field was captured, the stage was moved manually to a new field using fiducial landmarks to ensure a completely nonredundant evaluation.

### 2.7. Tissue Homogenates

Hippocampuses of five AD cases and four control cases were dissected from fresh-frozen brain. Samples were homogenized in 10 volumes of ice-cold sucrose buffer (0.32 M sucrose, 1 mM EDTA, 5 mM Tris-HCl, and pH 7.4) containing 0.2% Triton X-100 and a protease inhibitor cocktail (Sigma, USA). The homogenate was centrifuged at 10,000 ×g for 20 min. The supernatant was saved and the pellet was then resuspended in ice-cold sucrose buffer and homogenized again. The homogenate was solubilised for 1 hr at 4°C. Insoluble material was removed by centrifugation at 10,000 ×g for 30 min. The second supernatant was combined with the first as hippocampal solubilised fraction. After the estimation of protein concentration, aliquots of homogenates were immediately subjected to incubation with 4 × SDS-PAGE sample buffer (250 mM Tris-HCl, 8% SDS, 40% glycerol, 0.4 M DTT, 0.04% Bromophenol Blue, and pH 6.8) at 37°C for 20 min. Protein (50 *μ*g) from each sample was loaded and run on a 10% SDS-PAGE gel.

### 2.8. Immunoblot

Immunoblots were performed as described previously [27] and incubated with the affinity purified DPP10_789_ antibody (final concentration at 1.25 *μ*g/mL) or anti-*β*III-tubulin monoclonal antibody (final concentration 0.1 *μ*g/mL) at 4°C overnight. Bound antibodies were detected using the SuperSignal West Pico Chemiluminescent Substrate (Pierce, IL, USA). The membrane was exposed to X-OMAT diagnostic film (Kodak Scientific Light Systems, NY, USA) and developed on a Kodak X-OMAT 1000 Processor (Kodak Australasia, Pty. Ltd.).

### 2.9. Analytical Methods

The protein concentrations of brain homogenates were measured using the Bradford assay (BioRad, USA) according to the manufacturer's instructions. Levels of DPP10_789_ in brain homogenates were estimated by immunoblot using the Quantity One (BioRad, USA) image analysis software package and were adjusted by the level of neuron specific protein *β*-tubulin. An independent *t*-test was performed to compare the expression of DPP10_789_ between control and AD brains.

## 3. Results

### 3.1. Specificity of the DPP10_789_ Antibody

**Figure 1:**
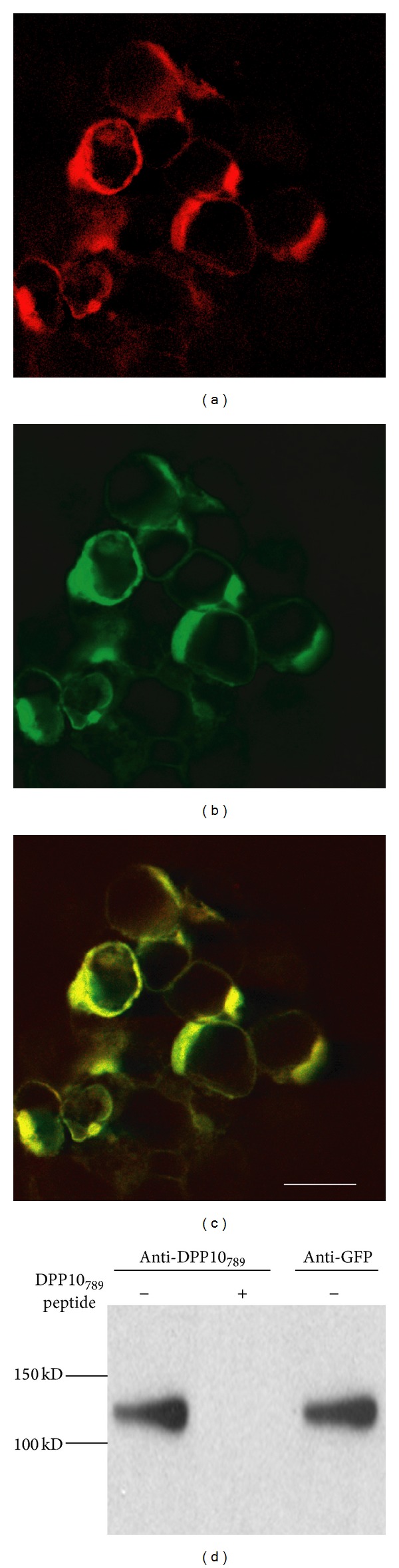
Anti-DPP10_789_ antibody characterization. The GFP-DPP10_789_ construct was transiently transfected into 293T cells. DPP10_789_ protein was stained with anti-DPP10_789_ antibody followed by Cy3 goat anti-rabbit IgG (a). The green GFP fluorescence (b) and the overlay of the Cy3 and the GFP images (c). Scale bar is 20 *μ*m. Membrane fractions were prepared and run on a 10% SDS-PAGE gel. The transferred membrane was immunoblotted with DPP10_789_ antibody. The DPP10_789_ antibody recognized a 120 kDa band corresponding to the full-length GFP-tagged DPP10_789_ ((d) far left lane). This 120 kDa band can be totally blocked with addition of the antigenic peptide for DPP10_789_ antibody in the blocking solution ((d) middle lane). The anti-GFP antibody detected the same band after the membrane was stripped and reprobed ((d) right lane).

To confirm the specificity of the anti-DPP10_789_ antibody, we used immunofluorescence and immunoblots to examine the 293T cells transfected with the GFP-DPP10_789_ construct (Figures 1(a)–1(d)). With immunofluorescence, all cells that expressed GFP fluorescence were detected by the anti-DPP10_789_ antibody (Figures 1(a), 1(b), and 1(c)), indicating the antibody specifically bound to recombinant GFP-DPP10_789_ chimeric protein. Our DPP10_789_ antibody was designed to detect the *N*-terminal sequence of DPP10_789_, while the anti-GFP antibody recognises the GFP protein. On immunoblot (Figure 1(d)), the DPP10_789_ antibody recognised a band around 120 kDa (Figure 1(d), far left lane), corresponding to the expected molecular mass of GFP-tagged DPP10_789_ and being consistent with our previous studies [27]. The 120 kDa band was also detected by the anti-GFP antibody (Figure 1(d), right lane) confirming that the band was the GFP-DPP10_789_ chimeric protein. Because 293T cells are derived from human kidney epithelial cells that do not express the brain derived DPP10_789_, no endogenous DPP10_789 _was detected by immunoblot. Thus these results confirmed that the DPP10_789_ antibody was specific to DPP10_789_.

### 3.2. DPP10_789_ in Control and AD Brains

**Figure 2:**
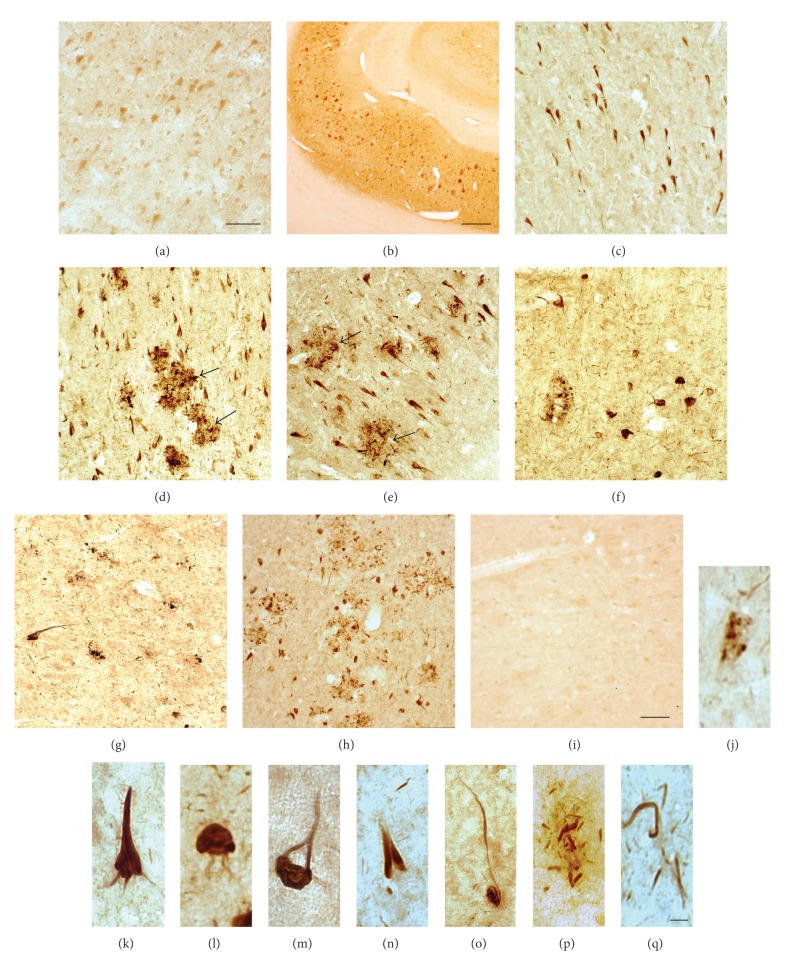
Immunostaining of DPP10_789_ in free-floating sections of control and AD brains. DPP10_789_ immunostaining in the CA1 region of a control brain (a), CA1 and subiculum region (b, c, and d), CA2 (e), inferior frontal cortex (f), cingulated cortex (g), inferior temporal cortex (h), and inferior temporal cortex section blocked with antigenic peptide (i) in AD brains. DPP10_789_ staining was found in granular structures in the neuronal soma (j), NFT with extensions into dendrites of pyramidal neurons (k), globose NFT (l), extracellular ghost tangle (m), flame shaped NFT (n), slender tangle of the subiculum with long extension into apical dendrite (o), plaque-associated dystrophic neurites (p), and neuropil threads (q). Scale bar is 200 *μ*m (b), 50 *μ*m (a, c–i), and 10 *μ*m (j–q). Arrows in (d) and (e) indicate staining in plaque-associated dystrophic neurites in AD brain.

**Figure 3:**
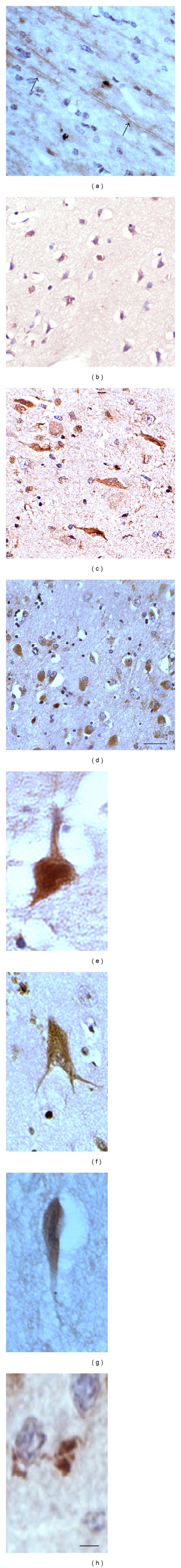
Immunostaining of DPP10_789_ in paraffin sections from control and AD brains. DPP10_789_ reactivity was observed in the frontal cortex of a control individual (a, b), and arrows indicate the surface staining of DPP10_789_ in dendrites. DPP10_789_ positive staining was increased in AD brain (c, d) and in NFTs (e–h). Scale bar is 50 *μ*m (a–d) and 10 *μ*m (e–h).

To document the cellular localisation of DPP10_789_ protein in human brains, we first immunostained free-floating brain sections from a selection of control cases and cases with various neurodegenerative diseases (Table 2). In control brains, positive DPP10_789_ staining was predominantly associated with neurons in the CA1 region of the hippocampus (Figures 2(a), 3(a), and 3(b)). The pyramidal cells were particularly well stained, with DPP10_789_ reactivity extending well into the distal portion of dendrites (Figures 3(a) and 3(b)).

In contrast, very strong punctate DPP10_789_ immunoreactivity was observed in AD brains especially in the hippocampal regions. Close examination revealed that neurofibrillary tangles and dystrophic neurites were robustly stained with the DPP10_789_ antibody. These positive structures were most abundant in the hippocampal region, where staining was most intense in the CA1 and subiculum areas (Figures 2(b)–2(e) and 2(h)). This immunoreactivity was completely abolished by the DPP10_789_ antigenic peptide (Figure 2(i)), and sections incubated with preimmunization serum omitting the DPP10_789_ antibody did not show any positive staining (data not shown), further indicating that the observed immunostaining was specific.

At high magnifications, three types of DPP10_789_ positive neurofibrillary tangle-like structures were observed: (1) granular staining in the neuronal soma (Figure 2(j)), resembling pretangle phosphotau aggregates; (2) condensed intraneuronal staining (Figures 2(k), 2(l), and 2(o)), resembling intraneuronal tau NFTs; (3) less condensed extracellular staining (Figures 2(m), 2(n), and 5(h)), resembling extraneuronal tau NFTs and ghost tangles. In addition, DPP10_789_ immunoreactivity was also detected significantly in dystrophic neurites surrounding amyloid plaques (Figures 2(d), 2(e), arrows, and 2(p)) and in neuropil threads (Figure 2(q)).

The DPP10_789_ positive tangles and dystrophic neurites were also observed in the frontal, temporal, entorhinal, and cingulate regions as summarized in Table 3. No DPP10_789_ positive staining was observed in the cerebellum.

Similar results were also observed in paraffin-embedded sections. DPP10_789_ immunostaining was detected on the cell body and surface of dendrites of the hippocampal region in a control case (Figures 3(a), arrows, and 3(b)). DPP10_789_ positive staining was also observed in NFTs and plaque-associated dystrophic neurites in AD brain (Figures 3(c)–3(h)). Thus the above results suggested that the increased DPP10_789 _staining was primarily accumulated in neurofibrillary tangles and dystrophic neurites.

### 3.3. DPP10_789_ in Other Tauopathies

**Figure 4:**
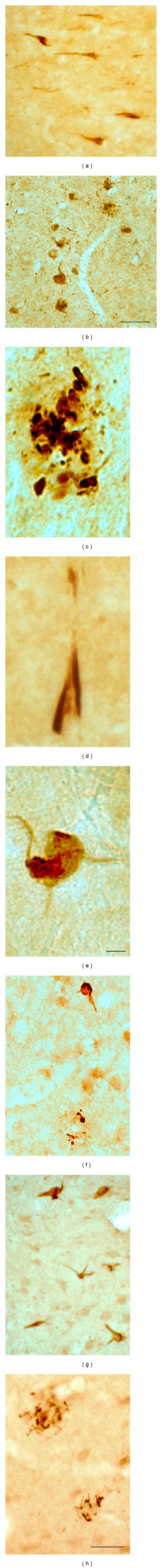
DPP10_789_ immunostaining in free-floating brain sections of other tauopathies. DPP10_789_ positive NFTs were observed in the hippocampus of FTLD (a, d and g) and PSP (f) and superior frontal brains sections from patients with DLBD (b, e). DPP10_789_ staining in plaque-associated dystrophic neurites in DLBD and PSP brains (c and h, resp.). Scale bar is 50 *μ*m (a, b, and f–h) and 10 *μ*m (c–e).

Because neurofibrillary tangle and dystrophic neurite pathologies also occur in neurodegenerative diseases other than AD, including PSP and FTLD and DLBD, these later cases were further examined. Some DPP10_789_ positive tangles and plaques were also detected in these cases (Figure 4). Table 3 summarizes the presence of DPP10_789_ positive tangles and dystrophic neurites. In the four DLBD cases examined, DPP10_789_ positive tangles and dystrophic neurites were also observed in temporal, hippocampal, and entorhinal cortex regions (Figures 4(b) and 4(e)). A few positive DPP10_789_ tangles and plaques in the hippocampal and entorhinal regions were observed in PSP cases (Figure 4(c)). Occasionally weak DPP10_789_ positive tangles and plaques were detected in some aged control brains.

### 3.4. DPP10_789_ Colocalizes with Tau Protein but Not with Amyloid-*β* in AD Brain

**Figure 5:**
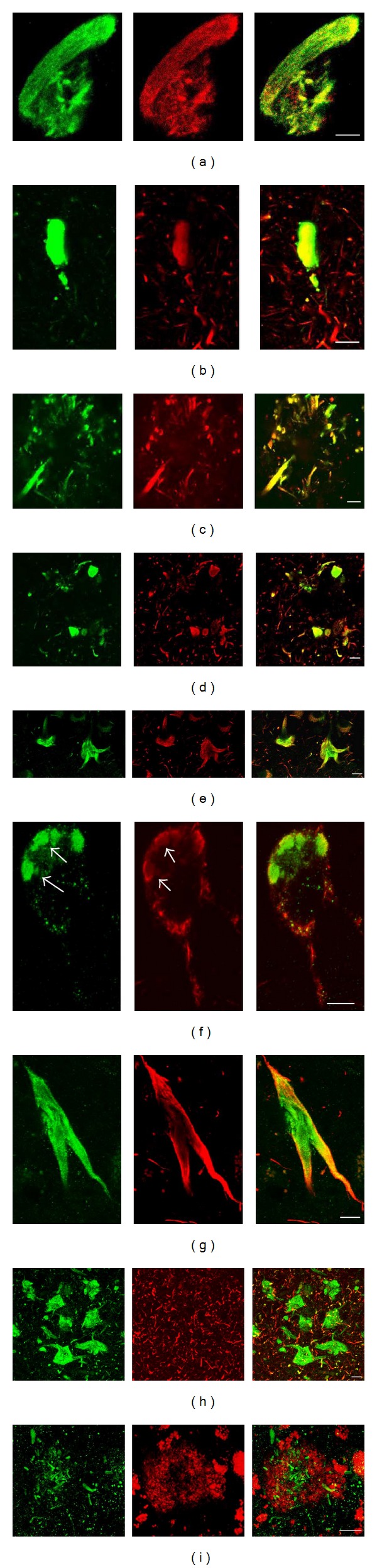
DPP10_789_ and tau colocalization in AD. Immunohistochemical colocalization of DPP10_789_ with phosphorylated tau (p-tau) in tangles and plaque-associated dystrophic neurites. DPP10_789_ (green) partially or fully colocalized with p-tau (red) in tangles (a, b, e, and g) and in plaque-associated dystrophic neurites (c, d). DPP10_789_ was also present in intracellular vesicle-like structures (f) where very weak staining for p-tau was present. DPP10_789_ immunoreactivity was also found in some tangles in the entorhinal region which were devoid of p-tau staining (h). DPP10_789_ (green) did not colocalize with amyloid-*β* plaques (red) in AD brain (i). Scale bar is 10 *μ*m. (a) and (c) used Tau2 ab and the remaining images are with AT8 ab.

Because tau protein is a major component of neurofibrillary tangles and dystrophic neurites, we examined if DPP10_789_ protein expression is associated with tau protein expression. We used the DPP10_789_ antibody and anti-tau antibody (AT8) specific for phospho-Ser202/Thr-205 to localise DPP10_789_ and phosphorylated tau (p-tau) by double immunofluorescence staining in the brains of AD. We observed robust colocalization of DPP10_789_ with p-tau in most tangles and dystrophic neurites (Figure 5). In neurofibrillary tangles, DPP10_789_ and p-tau were fully colocalized (Figures 5(b), 5(e), and 5(g)). In plaques, DPP10_789_ and p-tau were found in the larger sized dystrophic neurites (Figure 5(d)). DPP10_789_ was also present in intracellular vesicle-like structures (Figure 5(f)) where only weak p-tau staining was present. In the entorhinal region of the temporal cortex, some DPP10_789_ positive tangle-like structures were devoid of p-tau staining (Figure 5(h)), indicating that the DPP10_789_ antibody might be able to detect either early or late tangle stages—ghost tangles which are not detected by AT8. Double staining using the DPP10_789_ antibody and Tau2 antibody (detects both phosphorylated and nonphosphorylated tau) also showed colocalization of DPP10_789_ and tau in neurofibrillary tangles and dystrophic neurites (Figures 5(a) and 5(c)).

The other major pathological hallmark of AD is the neuritic amyloid plaques located in the extracellular space of the brain, comprised of an aggregated A*β* peptide core surrounded by dystrophic neurites. As the DPP10_789_ antibody showed strong immunoreactivity in plaque-associated dystrophic neurites, it was also examined if DPP10_789_ colocalized with A*β* peptide. Double immunofluorescence revealed that DPP10_789_ positive dystrophic neurites were mostly present in the A*β* core of senile plaques. While both DPP10_789_ and A*β* positive staining were observed within the same plaque region, they were not colocalized; the red A*β* staining was distributed as amyloid aggregation, with the green DPP10_789_ positive dystrophic neurites mingling randomly (Figure 5(i)).

**Figure 6:**
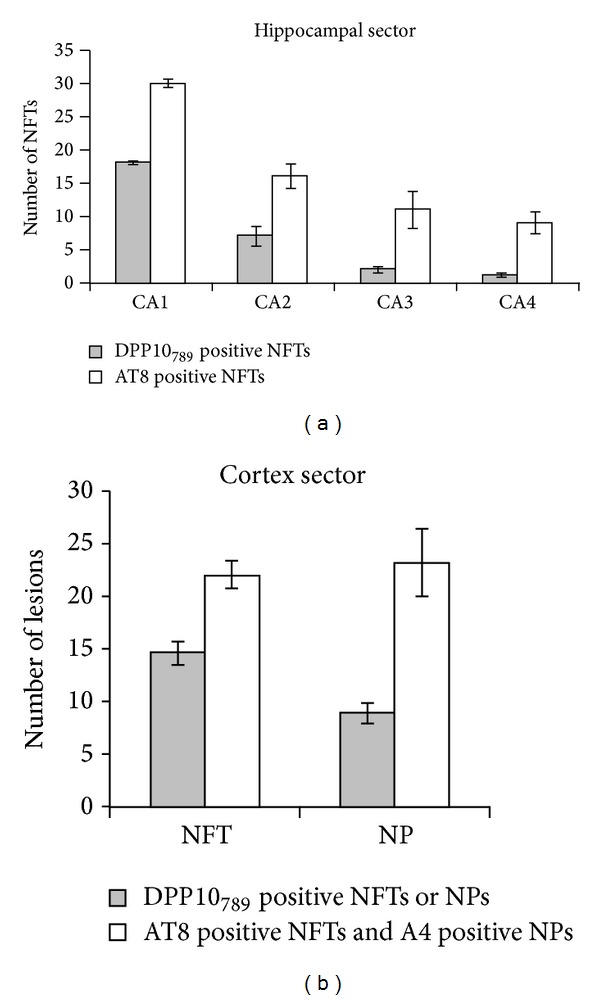
The regional distribution of DPP10_789_ positive NFTs and NPs compared with phosphotau positive NFTs and amyloid-*β* positive plaques. The adjacent free-floating sections of hippocampus or cortex were immunostained with DPP10_789_, AT8, or A4 antibody separately using the DAB reaction. (a) The distribution patterns of DPP10_789_ positive NFTs and tau positive NFTs in AD hippocampal sector. Three fields (0.01 mm^2^/field) per case from each region of Ammon's horn (CA1-4) were counted. (b) In the cortex sector of AD brain, the number of DPP10_789_ positive NFTs and NPs was compared with tau positive NFTs and amyloid-*β* positive NPs. Three fields (0.01 mm^2^/field) per case were counted in each of layers III to V. Bars reflect mean ± SE (*n* = 9; observations from 3 fields/case × 3 cases).

The intrahippocampal distribution of DPP10_789_ positive neurofibrillary tangles (NFTs) followed the pattern established for p-tau positive ones, with the greatest number of involved neurons lying within the CA1 region of Ammon's horn, followed by CA2, CA3, and CA4 (Figure 6(a)). In the neocortical region, staining was detected mainly in layers III–V. Approximately 67% of p-tau positive NTFs and 40% A*β* positive neuritic plaques (NPs) contained anti-DPP10_789_ immunoreactivity in these regions (Figure 6(b)).

### 3.5. Truncated DPP10_789_ Is Elevated in AD Tissue

**Figure 7:**
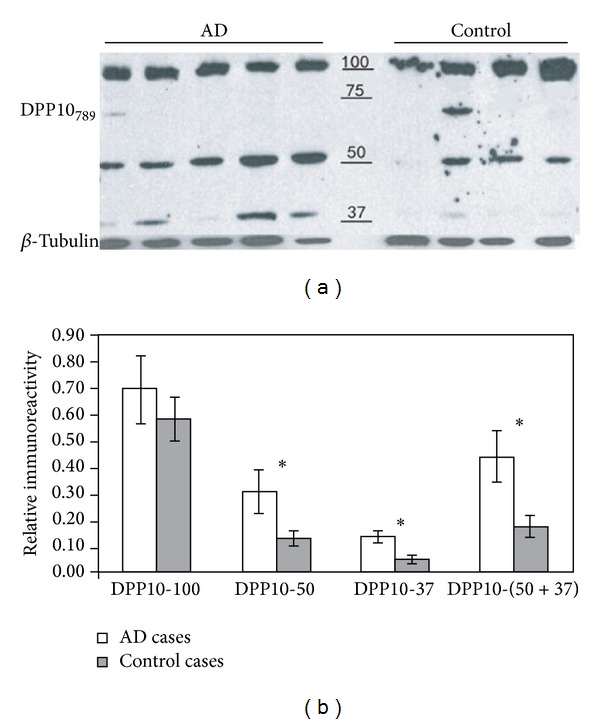
Truncated *N*-terminal DPP10_789_ levels were elevated in AD hippocampus relative to control hippocampus. 50 *μ*g of solubilised fractions prepared from fresh-frozen AD and control hippocampus was subjected to immunoblot analysis with DPP10_789_ antibody and class III *β*-tubulin antibody (a). Quantification of immunoblot data (b). The value of *y*-axis represents the relative immunoreactivity normalised to tubulin expression level. Bars represented mean ± SE of duplicate determination from five AD and four control hippocampal homogenates. “*” represented one-tailed *t*-test *P* ≤ 0.05%.

DPP10_789_ protein levels were analyzed in the hippocampus as it is a rich source of both NFTs and NPs. The DPP10_789_ Ab detected three major mobilities: 100 kDa, 50 kDa, and 37 kDa (Figure 7(a)) in immunoblots. The 100 kDa band represents the full-length DPP10_789_ protein [27]. Samples from both AD and control brains showed similar density of this band. The 50 kDa and 37 kDa bands observed are probably truncated forms of DPP10_789_ which are less than half the size of intact DPP10_789_ and have retained the *N*-terminus, suggesting that the DPP10_789_ has been proteolytically processed. Noticeably, AD brain samples had higher density bands of these two truncated forms compared to control samples. Quantitation of the immunoblot normalised to tubulin immunoreactivity confirmed that there was no significant difference in the 100 kDa intensity band between AD and control brains. However, the immunoreactive intensity of 50 kDa, 37 kDa, and 50 + 37 kDa was all significantly elevated in AD brain compared to control brain (*P* ≤ 0.05%.) (Figure 7(b)), indicating the possibility that *C*-terminal truncated DPP10_789_ may be aggregating and thus may be directly involved in the formation of neurofibrillary tangles and dystrophic neurites in AD brain.

## 4. Discussion

DPP10_789_ is a dipeptidyl peptidase-like protein that together with Kv-channel interacting proteins (KChIPs) forms multiprotein complexes that underlie subthreshold A-type currents (*I*_*SA*_) in neuronal somatodendritic compartments [28]. This is the first study to assess the expression of DPP10_789_ in postmortem brain samples in AD and other major tauopathies. Using immunohistochemistry we reveal predominant neuronal staining of DPP10_789_ throughout the neocortex and subcortical grey matters with high expression in the pyramidal cells in control brain tissue. In contrast, in AD brains, robust DPP10_789_ reactivity was also detected in neurofibrillary tangles and plaque-associated dystrophic neurites, most of which colocalized with the hyperphosphorylated Ser-202/Thr-205 tau epitope. DPP10_789_ positive neurofibrillary tangles and plaque-associated dystrophic neurites also appeared in other neurodegenerative diseases such as FTLD, DBLB, and PSP. Occasional DPP10_789_ positive neurofibrillary tangles and neurites were seen in some aged control brains. Furthermore using a quantitative analysis we showed that truncated DPP10_789_ fragments increased significantly in AD brains compared to control brains. These results provide the first evidence that DPP10_789_ may be involved in the pathology of AD and other neurodegenerative diseases.

Our immunohistochemistry studies revealed that in control brains DPP10_789_ immunoreactivity is located in the cell body of neurons throughout the grey matter of cerebral cortex and axonal fibre tracts of white matter (Figures 2(a) and 3(a)), suggesting that DPP10_789_ protein is widely distributed in the neuronal cells which is consistent with the expression of DPP10 mRNA reported in rat brain [28]. In human there are potentially four DPP10 mRNA isoforms which all have unique amino acid sequences in the *N*-terminal intracellular domain but identical *C*-terminal extracellular domains [31]. Multiple DPP10 isoforms have been observed by both Northern blot and reverse transcriptase PCR in both human brain and pancreas; however it is not known which transcript on the Northern blot represents DPP10_789_ or whether any of these mRNA isoforms are translated into different forms of DPP10 protein* in vivo* [27, 31–33]. Takimoto et al. demonstrated the presence of four DPP10 mRNA isoforms in human brain. These isoforms are different only at the *N*-terminal amino acid sequence. When the different *N*-terminal forms of DPP10 were co-expressed with the Kv4.3 channel there were no differences in the gating ability of the channel [31]. In their study DPPYd is equivalent to DPP10_789_ and this form was also detected in rat brain. Interestingly Takimoto et al. were unable to detect DPP10_796_ in the adrenal gland and pancreas of humans or rats. In our laboratory we have found it extremely difficult to use reverse transcriptase PCR to amplify the other DPP10 isoforms (data not shown) from either postmortem brain tissue or neuronal cell lines; therefore these mRNA transcripts and thus protein isoforms appear to be present in very low amounts in human tissues.

Throughout the cortex of AD cases, although the background distribution pattern of DPP10_789_ is similar to controls, it was the neurofibrillary tangles and plaque-associated dystrophic neurites that, by virtue of their intense DPP10_789_ staining, stand out compared to controls (some aged controls also had a few DPP10_789_ tangles). Our data also revealed that the DPP10_789_ positive tangles and plaque-associated dystrophic neurites are distributed mainly in layers III–V pyramidal cells of the neocortex and CA1 and CA2 pyramidal cells of the hippocampus in AD brains, consistent with the distribution of Kv4.2 and Kv4.3 subunits in brain [34]. DPP10 interacts with both Kv4.2 and Kv4.3 subunits [17, 35]. Additionally, Kv4 channels play important roles particularly in regulating neuronal membrane excitability; therefore these results indicate that deposition of DPP10_789_ protein in these neurons may contribute to loss of channel function and lead to neuronal dysfunction and cell death in AD. Double immunofluorescence staining revealed that most of the DPP10_789_ positive tangles were colocalized with the hyperphosphorylated phospho-Ser-202/Thr-205 tau epitope. In addition, the intrahippocampal distribution of DPP10_789_ positive NFTs followed the pattern established for phospho-Ser-202/Thr-205 tau positive NFTs; however the amount of DPP10_789_ positive NFTs was always less than that of phospho-Ser-202/Thr-205 tau (Figure 6(a)). This implies that, while AT8 can detect all six isoforms of human tau protein in brain [36], DPP10_789_ may only be present in tangles containing a subset of the six isoforms. On the other hand, in the entorhinal cortex region, some DPP10_789_ positive tangles were devoid of either AT8 (Figure 5(h)) or Tau2 (data not shown) antibody staining, indicating that DPP10_789_ did not always cooccur with tau in tangles and it may on its own be involved with tangle formation without tau involvement. It is also possible that our DPP10_789_ antibody can detect the late stage of tangles—the ghost tangles which have lost the phospho-Ser-202/Thr-205 tau epitope recognised by the AT8 antibody [37, 38]. Recently Lace et al. reported that the RD3 antibody that specifically recognises the 3-repeat tau shows an increased immunoreactivity in late-state ghost tangles [39]. Do RD3 positive tangles colocalize with DPP10_789_ positive staining? Which tau isoform may be involved in DPP10_789_ positive tangle formation? To answer these questions, further study needs to be done by using different tau antibodies specific to different tau isoforms.

Tau is a microtubule-associated protein and predominantly found in axons [40–42]. The best known functions of tau are the stabilization of microtubules and the regulation of axonal transport [43, 44]. Recently it was reported that tau has also been localized to dendrites under physiological conditions and at much lower levels [45]. In addition to its interaction with tubulin, tau binds to other partners such as the tyrosine protein kinase Fyn, dynactin, and postsynaptic density protein 95 (PSD95) [45–47]. Both tau-Fyn and tau-PSD95 interactions are involved in the complex formation of NMDA receptors with PSD95, which is required for excitotoxic downstream signalling, suggesting that the distribution of tau in dendrites is pivotal to healthy neurons [45, 47]. Noticeably, the intracellular distributions of Kv4 channels are also predominant in dendrites [34]. Besides, it has been reported that PSD95 has physical interactions with Kv4.2 [48, 49], suggesting that PSD95 may be involved in forming complexes between Kv4.2 and signalling molecules. Because the dendritic A-type potassium current strongly influences the induction of long-term potentiation also by NMDA receptors [50, 51], it is possible that there exist some signalling connections between tau, Fyn, PSD95, and Kv4. As a Kv4 channel associated protein, DPP10 also has the potential to be involved in these connections. However, so far there is no direct evidence showing that tau interacts with Kv4, and one possible explanation is that these protein interactions may be transient or unstable; thus it is difficult to detect the interactions using immunoprecipitation, immunostaining techniques, or other proteomic based methods.

DPP10_789_ positive immunoreactivity was also observed in dystrophic neurites in A*β* aggregated senile plaques. It was found that DPP10_789_ positive dystrophic neurites did not colocalize but mingled with the A*β* deposition core of the senile plaques. This is consistent with the fact that plaques are composed of extracellular aggregated A*β*, while DPP10_789_, according to our studies, is distributed intracellularly or in the membrane [26, 27]. In addition, we have observed that the number of DPP10_789_ positive neurofibrillary tangles and plaque-associated dystrophic neurites present in AD is much more than that observed in other tauopathies (Table 3). As the accumulation of A*β* is a primary event driving AD pathogenesis, our finding indicates that DPP10_789_ may be involved some way in the pathogenic process. So far, the mechanisms by which A*β* mediates neurotoxicity and initiates the degenerative processes of AD are still not clear. Recent studies revealed that A*β* inhibits the dendritic A-type K+ current in outside-out patches excised from distal dendrites of hippocampal CA1 pyramidal neurons, causing increases in back-propagating dendritic action-potential amplitude and associated Ca^2+^ influx. These results suggest that the sustained increased dendritic Ca^2+^ influx, which results from A*β* deposition in dendritic arborisation that induces persistent inhibition of the A-type K+ current, may cause a loss of Ca^2+^ homeostasis in dendrites of hippocampal neurons, an initiating event of synaptic failure and neurodegeneration [52]. Whether DPP10_789_ is involved in this procedure needs further study.

The quantitative immunoblot on human brain samples showed that naturally* in vivo* two truncated forms of DPP10_789_ are observed and the levels of these increase in AD brains compared to control brains, which has led us to hypothesise that the truncated forms may form aggregates in NFTs and dystrophic neurites. As there was no change in the full-length form of DPP10_789_ between control and AD patients, it is assumed that if there is any increase in protein production in AD, the newly formed protein is likely to be rapidly degraded. DPP10_789_ is a glycosylated transmembrane protein with a large extracellular *C*-terminal domain and a short intracellular *N*-terminal domain. The presence of DPP10_789_ in NFTs and dystrophic neurites but not amyloid protein aggregated plaques suggests that DPP10_789_ is confined to the neuronal cytoplasm. These observations suggest the possible aberrant trafficking of DPP10_789_ in degenerating neurons.

DPP10 belongs to DPP4 gene family, some members of which have been reported to have multiple functions. Previous* in vitro* studies showed that DPP4 and FAP have roles in the cell-extracellular matrix interactions and apoptosis, and these functions are independent of catalytic activity [53]. It is likely that most protein-protein interactions would occur on the *β*-propeller domains of these proteins [54]. With 47% amino acid similarity to DPP4, it is possible that DPP10, apart from associating with Kv4 channel complex through its single transmembrane domain [55], may also interact with other proteins and ligands via either its extracellular *β*-propeller domain or its intracellular domain and these interactions may control its biological function in normal and pathological conditions. Recently Lin et al. elegantly demonstrated that the highly related DPP6 interacts with filopodia-associated myosin and fibronectin within the extracellular matrix to play a role in hippocampal synaptic development [56]. In addition, DPP6 has been shown to interact with prion protein to mediate potassium channels and neuronal excitability [57].

In summary this paper reports the first evidence that DPP10_789_ protein is present in NFTs and dystrophic neurites in AD. Interestingly, DPP10_789_ protein colocalizes with hyperphosphorylated tau protein but not with A*β*. Moreover in neurodegenerative cases other than AD where neuronal tau pathology occurred, the same regions showed intense staining for DPP10_789_. In addition this paper provides initial evidence that, as with other proteins involved in neurodegenerative diseases, DPP10_789_ *C*-terminal truncation may lead to mistrafficking and aggregation of DPP10_789_ protein but at this point it is unclear whether this occurrence contributes to AD pathology or is a result of AD pathology. Together this data suggests that DPP10 may play a significant role in the neurodegenerative process observed in AD and further investigation into this role and its interaction with tau is warranted.

## Figures and Tables

**Table 1:** Primary antibodies used in this study.

Antibody/antiserum	Species	Epitope	Source	Dilution
DPP10_789_ (polyclonal)	Rabbit	DPP10_789_ *N*-terminal intracellular domain	This lab	1 : 100

Anti-GFP (polyclonal)	Rabbit	GFP	Invitrogen, USA	1 : 3000

AT8	Mouse	Specific for tau phosphorylation at Ser 202 and/or Thr 205	Innogenetics, Belgium	1 : 1000

Tau2	Mouse	Phosphorylated and nonphosphorylated tau	Novocastra, UK	1 : 1000

A4	Mouse	*β*-Amyloid	Professor Colin Masters, Melbourne University	1 : 250

Anti-*β*III-tubulin monoclonal Ab	Mouse	*C*-Terminus of *β*III-tubulin	Promega, USA	1 : 10000

**Table 2:** Human cases analysed in this study.

Brain Bank number	Gender	Age	Postmortem delay (h)	Neuropathological diagnosis
SA0123	M	80	21	AD
SA0126	F	79	31	AD
SA0134	F	69	23	AD
SA0143	M	84	15	AD
SA0148	M	81	14	AD
SA0129	F	88	9	Early AD

SA0087	M	71	15.5	FTLD

SA0069	M	69	31	DLBD
SA0079	F	82	31	DLBD
SA0063	F	81	7	DLBD
SA0094	M	74	24	DLBD

SA0035	F	72	28	PSP
SA0043	F	69	10	PSP
SA0106	M	66	29	PSP
SA0136	F	80	20	PSP

SA0010	M	58	14	Control
SA0013	M	64	23	Control
SA0021	F	72	7	Control
SA0030	M	69	48	Control
SA0031	F	61	8	Control

AD: Alzheimer's disease, DLBD: diffuse Lewy's body disease, FTLD: frontotemporal lobar degeneration, PSP: progressive supranuclear palsy, F: female, and M: male.

**Table 3:** Summary of the DPP10_789_ positive staining in NFT and plaque-associated dystrophic neurites observed in control and neurodegenerative disease cases.

	Case number	Neuropathological diagnosis	Frontal cortex		Temporal cortex		Hippocampus		Entorhinal cortex		Cingulate cortex	
			Tangle	Plaque	Tangle	Plaque	Tangle	Plaque	Tangle	Plaque	Tangle	Plaque
1	SA0123	AD	−	+++	+++	+++	+++	+++	+++	+++	++	+++
2	SA0126	AD	+++	++	+++	+++	++	+++	+++	++	++	++
3	SA0134	AD	+	+++	−	+++	+++	++	N/A	N/A	+	+++
4	SA0143	AD	+	+	+++	++	+++	+++	+++	+++	+	+
5	SA0148	AD	+++	+++	−	+++	+++	+++	+++	+++	+++	+++
6	SA0129	Early AD	+	+	+	+	+++	++	+++	+	−	−

7	SA0069	DLBD	++	++	−	+++	++	++	+++	+++	+	++
8	SA0079	DLBD	−	−	−	−	−	++	−	−	−	−
9	SA0094	DLBD	−	−	+++	+++	+	++	++	++	+	+
10	SA0063	DLBD	−	−	+	−	+	+	−	−	−	−

11	SA0087	FTLD	−	−	−	−	+++	+	++	−	−	−

12	SA0035	PSP	−	−	+	−	+	−	N/A	N/A	−	−
13	SA0043	PSP	+	+	+	+	++	+	++	++	+	−
14	SA0106	PSP	−	−	−	−	−	−	++	N/A	−	−
15	SA0136	PSP	−	−	−	+	−	++	+++	+	−	−

16	SA0010	Control	+	−	−	+	−	−	−	−	−	+
17	SA0013	Control	−	−	−	−	+	−	−	−	−	−
18	SA0021	Control	−	−	−	−	+	−	+	−	−	−
19	SA0030	Control	−	−	+	−	−	−	N/A	N/A	+	+
20	SA0031	Control	−	−	−	−	N/A	N/A	N/A	N/A	−	−

The number of lessons was counted at ×200 magnification under an Olympus BH-2 light microscope (Tokyo, Japan).

N/A: sample not available; −: negative; +: 1–5 lesions/field; ++: 5–10 lesions/field; +++: >10 lesions/field.

## Abbreviations

AD: Alzheimer's disease
A*β*: Amyloid-*β* peptide
DAB: 3-3-Diaminobenzidine-4 HCl/H_2_O_2_
DLBD: Diffuse Lewy's body disease
DP: Dipeptidyl peptidase
FTLD: Frontotemporal lobar degeneration
GFP: Green fluorescent protein
Kv channel: Voltage-gated potassium channel
KChIP: Kv channel interacting protein
NFTs: Neurofibrillary tangles
NPs: Neuritic plaques
PSP: Progressive supranuclear palsy.

## References

[1] Masters CL, Cappai R, Barnham KJ, Villemagne VL (2006). Molecular mechanisms for Alzheimer’s disease: implications for neuroimaging and therapeutics. *Journal of Neurochemistry*.

[2] Selkoe DJ (2002). Alzheimer’s disease is a synaptic failure. *Science*.

[3] Hardy J (2006). A hundred years of Alzheimer's disease research. *Neuron*.

[4] Goate A, Chartier-Harlin M-C, Mullan M (1991). Segregation of a missense mutation in the amyloid precursor protein gene with familial Alzheimer’s disease. *Nature*.

[5] Thinakaran G, Sisodia SS (2006). Presenilins and Alzheimer disease: the calcium conspiracy. *Nature Neuroscience*.

[6] Corder EH, Saunders AM, Strittmatter WJ (1993). Gene dose of apolipoprotein E type 4 allele and the risk of Alzheimer’s disease in late onset families. *Science*.

[7] Selkoe DJ (2004). Cell biology of protein misfolding: the examples of Alzheimer’s and Parkinson’s diseases. *Nature Cell Biology*.

[8] Oddo S, Billings L, Kesslak JP, Cribbs DH, LaFerla FM (2004). A*β* immunotherapy leads to clearance of early, but not late, hyperphosphorylated tau aggregates via the proteasome. *Neuron*.

[9] Etcheberrigaray R, Ito E, Oka K, Tofel-Grehl B, Gibson GE, Alkon DL (1993). Potassium channel dysfunction in fibroblasts identifies patients with Alzheimer disease. *Proceedings of the National Academy of Sciences of the United States of America*.

[10] de Silva HA, Aronson JK, Grahame-Smith DG, Jobst KA, Smith AD (1998). Abnormal function of potassium channels in platelets of patients with Alzheimer’s disease. *The Lancet*.

[11] Shah NH, Aizenman E (2014). Voltage-gated potassium channels at the crossroads of neuronal function, ischemic tolerance, and neurodegeneration. *Translational Stroke Research*.

[12] Wu X, Hernandez-Enriquez B, Banas M, Xu R, Sesti F (2013). Molecular mechanisms underlying the apoptotic effect of KCNB1 K^+^ channel oxidation. *The Journal of Biological Chemistry*.

[13] Yamamoto K, Ueta Y, Wang L (2011). Suppression of a neocortical potassium channel activity by intracellular amyloid-*β* and its rescue with homer1a. *Journal of Neuroscience*.

[14] Jerng HH, Pfaffinger PJ, Covarrubias M (2004). Molecular physiology and modulation of somatodendritic A-type potassium channels. *Molecular and Cellular Neuroscience*.

[15] Birnbaum SG, Varga AW, Yuan L-L, Anderson AE, Sweatt JD, Schrader LA (2004). Structure and function of Kv4-family transient potassium channels. *Physiological Reviews*.

[16] Nadal MS, Ozaita A, Amarillo Y (2003). The CD26-related dipeptidyl aminopeptidase-like protein DPPX is a critical component of neuronal A-type K^+^ channels. *Neuron*.

[17] Jerng HH, Kunjilwar K, Pfaffinger PJ (2005). Multiprotein assembly of Kv4.2, KChIP3 and DPP10 produces ternary channel complexes with ISA-like properties. *Journal of Physiology*.

[18] Ikeda M, Dewar D, McCulloch J (1991). Preservation of [^125^I]galanin binding sites despite loss of cholinergic neurons to the hippocampus in Alzheimer’s disease. *Brain Research*.

[19] Ramsden M, Henderson Z, Pearson HA (2002). Modulation of Ca^2+^ channel currents in primary cultures of rat cortical neurones by amyloid *β* protein (1–40) is dependent on solubility status. *Brain Research*.

[20] Ramsden M, Plant LD, Webster NJ, Vaughan PFT, Henderson Z, Pearson HA (2001). Differential effects of unaggregated and aggregated amyloid *β* protein (1–40) on K^+^ channel currents in primary cultures of rat cerebellar granule and cortical neurones. *Journal of Neurochemistry*.

[21] Buxbaum JD, Choi E-K, Luo Y (1998). Calsenilin: a calcium-binding protein that interacts with the presenilins and regulates the levels of a presenilin fragment. *Nature Medicine*.

[22] Choi E-K, Zaidi NF, Miller JS (2001). Calsenilin is a substrate for caspase-3 that preferentially interacts with the familial Alzheimer’s disease-associated C-terminal fragment of presenilin 2. *The Journal of Biological Chemistry*.

[23] Jo D-G, Lee J-Y, Hong Y-M (2004). Induction of pro-apoptotic calsenilin/DREAM/KChIP3 in Alzheimer’s disease and cultured neurons after amyloid-*β* exposure. *Journal of Neurochemistry*.

[24] Coles B, Wilton LAK, Good M, Chapman PF, Wann KT (2008). Potassium channels in hippocampal neurones are absent in a transgenic but not in a chemical model of Alzheimer’s disease. *Brain Research*.

[25] Liu D, Pitta M, Lee J-H (2010). The KATP channel activator diazoxide ameliorates amyloid-*β* and Tau pathologies and improves memory in the 3xTgAD mouse model of Alzheimer’s disease. *Journal of Alzheimer’s Disease*.

[26] Chen T, Smyth D, Abbott CA (2004). CD26. *Journal of Biological Regulators and Homeostatic Agents*.

[27] Chen T, Ajami K, McCaughan GW, Gai W-P, Gorrell MD, Abbott CA (2006). Molecular characterization of a novel dipeptidyl peptidase like 2-short form (DPL2-s) that is highly expressed in the brain and lacks dipeptidyl peptidase activity. *Biochimica et Biophysica Acta*.

[28] Jerng HH, Lauver AD, Pfaffinger PJ (2007). DPP10 splice variants are localized in distinct neuronal populations and act to differentially regulate the inactivation properties of Kv4-based ion channels. *Molecular and Cellular Neuroscience*.

[29] McNicholas K, Chen T, Abbott CA (2009). Dipeptidyl peptidase (DP) 6 and DP10: novel brain proteins implicated in human health and disease. *Clinical Chemistry & Laboratory Medicine*.

[30] Abbott CA, McCaughan GW, Levy MT, Church WB, Gorrell MD (1999). Binding to human dipeptidyl peptidase IV by adenosine deaminase and antibodies that inhibit ligand binding involves overlapping, discontinuous sites on a predicted *β* propeller domain. *European Journal of Biochemistry*.

[31] Takimoto K, Hayashi Y, Ren X, Yoshimura N (2006). Species and tissue differences in the expression of DPPY splicing variants. *Biochemical and Biophysical Research Communications*.

[32] Allen M, Heinzmann A, Noguchi E (2003). Positional cloning of a novel gene influencing asthma from Chromosome 2q14. *Nature Genetics*.

[33] Qi SY, Riviere PJ, Trojnar J, Junien J-L, Akinsanya KO (2003). Cloning and characterization of dipeptidyl peptidase 10, a new member of an emerging subgroup of serine proteases. *Biochemical Journal*.

[34] Vacher H, Mohapatra DP, Trimmer JS (2008). Localization and targeting of voltage-dependent ion channels in mammalian central neurons. *Physiological Reviews*.

[35] Zagha E, Ozaita A, Chang SY (2005). DPP10 modulates Kv4-mediated A-type potassium channels. *The Journal of Biological Chemistry*.

[36] Gendron TF (2009). The role of tau in neurodegeneration. *Molecular Neurodegeneration*.

[37] Braak E, Braaak H, Mandelkow E-M (1994). A sequence of cytoskeleton changes related to the formation of neurofibrillary tangles and neuropil threads. *Acta Neuropathologica*.

[38] Ikeda K, Haga C, Oyanagi S, Iritani S, Kosaka K (1992). Ultrastructural and immunohistochemical study of degenerate neurite-bearing ghost tangles. *Journal of Neurology*.

[39] Lace G, Savva GM, Forster G (2009). Hippocampal tau pathology is related to neuroanatomical connections: an ageing population-based study. *Brain*.

[40] Aronov S, Aranda G, Behar L, Ginzburg I (2001). Axonal tau mRNA localization coincides with tau protein in living neuronal cells and depends on axonal targeting signal. *Journal of Neuroscience*.

[41] Utton MA, Connell J, Asuni AA (2002). The slow axonal transport of the microtubule-associated protein tau and the transport rates of different isoforms and mutants in cultured neurons. *Journal of Neuroscience*.

[42] Konzack S, Thies E, Marx A, Mandelkow E-M, Mandelkow E (2007). Swimming against the tide: mobility of the microtubule-associated protein tau in neurons. *Journal of Neuroscience*.

[43] Kosik KS (1993). The molecular and cellular biology of tau. *Brain Pathology*.

[44] Götz J, Ittner LM, Kins S (2006). Do axonal defects in tau and amyloid precursor protein transgenic animals model axonopathy in Alzheimer’s disease?. *Journal of Neurochemistry*.

[45] Ittner LM, Ke YD, Delerue F (2010). Dendritic function of tau mediates amyloid-*β* toxicity in alzheimer’s disease mouse models. *Cell*.

[46] Magnani E, Fan J, Gasparini L (2007). Interaction of tau protein with the dynactin complex. *The EMBO Journal*.

[47] Ittner LM, Götz J (2011). Amyloid-*β* and tau—a toxic pas de deux in Alzheimer’s disease. *Nature Reviews Neuroscience*.

[48] Wong W, Newell EW, Jugloff DGM, Jones OT, Schlichter LC (2002). Cell surface targeting and clustering interactions between heterologously expressed PSD-95 and the Shal voltage-gated potassium channel, Kv4.2. *The Journal of Biological Chemistry*.

[49] Wong W, Schlichter LC (2004). Differential recruitment of Kv1.4 and Kv4.2 to lipid rafts by PSD-95. *The Journal of Biological Chemistry*.

[50] Hoffman DA, Magee JC, Colbert CM, Johnston D (1997). K^+^ channel regulation of signal propagation in dendrites of hippocampal pyramidal neurons. *Nature*.

[51] Lei Z, Deng P, Li Y, Xu ZC (2010). Downregulation of Kv4.2 channels mediated by NR2B-containing NMDA receptors in cultured hippocampal neurons. *Neuroscience*.

[52] Chen C (2005). *β*-amyloid increases dendritic Ca^2+^ influx by inhibiting the A-type K^+^ current in hippocampal CA1 pyramidal neurons. *Biochemical and Biophysical Research Communications*.

[53] Chen T, Ajami K, McCaughan GW, Gorrell MD, Abbott CA, Hildebrandt M (2003). Dipeptidyl peptidase IV gene family: the DPIV family. *Advances in Experimental Medicine and Biology*.

[54] Gorrell MD (2003). First bite. *Nature Structural Biology*.

[55] Li H-L, Qu Y-J, Yi CL (2006). DPP10 is an inactivation modulatory protein of Kv4.3 and Kv1.4. *American Journal of Physiology*.

[56] Lin L, Sun W, Throesch B (2013). DPP6 regulation of dendritic morphogenesis impacts hippocampal synaptic development. *Nature Communications*.

[57] Mercer RC, Ma L, Watts JC (2013). The prion protein modulates A-type K^+^ currents mediated by Kv4.2 complexes through dipeptidyl aminopeptidase-like protein 6. *The Journal of Biological Chemistry*.

